# Chronic diseases and self-rated health disparity between urban and rural residents in China

**DOI:** 10.1371/journal.pone.0324287

**Published:** 2025-05-21

**Authors:** Yu Wang, Li Liu

**Affiliations:** 1 College of Public Health, Chongqing Medical University, Chongqing, China; 2 Yongchuan Hospital of Chongqing Medical University, Chongqing, China; Tribhuvan University Institute of Medicine, NEPAL

## Abstract

This study investigates the significant disparities between urban and rural areas in China, particularly in terms of health status, which are driven by economic inequality and the uneven distribution of healthcare resources. Chronic diseases are a major threat to the health of Chinese residents, and this study explores how these diseases contribute to the disparity in self-rated health between urban and rural populations. Using data from the 2021 Chinese General Social Survey, various data analysis methods, including descriptive, regression, and decomposition analyses, were employed. The results reveal substantial disparities in self-rated health between urban and rural residents, with chronic diseases playing a significant role in explaining these disparities. Approximately 39% of the urban-rural disparity in self-rated health can be explained by differences in chronic disease prevalence, with additional factors such as age, socio-economic status, social participation, and sleep quality also contributing. This study identified the correlation between chronic diseases and the disparity in self-rated health, and limitations may arise from the use of self-reported health and the complexity of urban-rural health disparities. The findings suggest that the urban-rural disparity in chronic diseases is the primary driver of the health disparity, and that policymakers should focus on improving health education, promoting chronic disease prevention and management, and emphasizing preventive healthcare in rural areas.

## Introduction

Health is related to quality of life, national security and social stability, and is the basis for human survival and development. It plays an important role in national economic development and is a basic condition for economic and social development [1]. In recent years, with social development and improved living standards, the health status of Chinese residents has significantly improved [2,3], and the average life expectancy has continued to increase. However, the longstanding urban-rural dual structure has led to disparities in opportunities for accumulating health-related human capital. According to the results of the National Health and Wellness Commission’s Health Literacy Monitoring Points for Residents in 2023, the health literacy level of urban residents nationwide was 33.25% in 2023, and that of rural residents was 26.23% [4]. The development of health literacy remains uneven between urban and rural areas. The issue of health disparities among different population groups has attracted widespread attention in both developed and developing countries. Health disparities are defined as systematic disparities in health outcomes between different population groups [5,6]. In this study, we used the Self-Rated Health (SRH) as an indicator of the health status of the population. The SRH not only reflects the health status of an individual, but also integrates subjective and objective aspects of health status [7].

Most studies have found that urban residents have better health than rural residents. It has been found that rural Americans are more likely to report risk factors associated with poorer health [8,9], and residents living in rural areas typically have poorer health than their urban counterparts [10]. In Brazil, although rural residents have better mortality indicators, urban residents report better self-rated health [11]. Kaczmarek’s study found that middle-aged women in urban areas have a higher quality of life than those in rural areas [12]. In fact, rural communities around the world are not only in poorer health than their urban counterparts, but also have more problems accessing health care [13]. The mechanisms that contributed to the urban-rural health disparity were multifactorial and complex. The management of multiple chronic diseases requires more health-care interactions, and the lack of access to health-care services in rural areas creates significant barriers to the management of chronic diseases [14]. In this study, urban areas refer to provincial capitals, prefecture-level cities, and their subordinate urban districts, while rural areas refer to townships and the villages under their jurisdiction.

In conclusion, with the transition of diseases, chronic diseases have been recognized as major threats to human health [15]. In 2016, non-communicable diseases caused 41 million deaths globally, accounting for 71% of global mortality. Studies have shown that chronic diseases are closely related to poor SRH [16], with some researchers finding that patients with chronic obstructive pulmonary disease (COPD) perceive their health status more negatively [17]. As age increases, the incidence of chronic diseases rises, and the prevalence of multimorbidity also increases, a trend that is expected to continue [18,19]. In China, studies show that chronic diseases have become a major threat to residents’ health, accounting for about 70% of the total disease burden [20], and have a significant negative impact on health-related quality of life [21].

However, although studies generally suggest that chronic diseases affect individuals’ SRH, the relationship between chronic diseases and SRH varies across different regions, cultures, and socioeconomic backgrounds. Few studies have specifically explored whether the disparities in chronic diseases between urban and rural residents are associated with SRH disparities, and to what extent chronic diseases contribute to the urban-rural SRH disparity. Some studies have indicated that chronic diseases affect SRH through physical symptoms, psychological distress, and functional limitations, and these factors may be exacerbated by differences in healthcare accessibility and social determinants of health [22]. To extend the current knowledge in explaining the urban–rural disparity in SRH among residents in China from the perspective of chronic diseases, this study aimed to address three questions: (1) to measure the disparities in SRH between urban and rural residents; (2) to examine the association between chronic diseases and residents’ SRH, and (3) to explore the extent to which the disparities in SRH between urban and rural residents can be explained by chronic disease disparities. Through these explorations, the aim is to gain a deeper understanding of the impact of chronic diseases on urban-rural health disparities, providing new insights for public health interventions to reduce inequalities.

## Materials and methods

### Data

The data used in this study came from the China General Social Survey (CGSS) conducted by the China Survey and Data Centre (NSRC) of Renmin University of China in 2021. The CGSS, which began in 2003, is China’s earliest nationwide, comprehensive, and continuous academic survey project. The survey adopts a multistage stratified sampling method, with Chinese citizens aged 18 and above as the target respondents, and a face-to-face interview model to conduct a continuous cross-section survey of information at multiple levels of society, community, household, and individual, with a total of 100 counties plus 5 major metropolitan cities, which is a good representation of the country. CGSS2021 is the latest publicly available data covering 320 communities in 19 provinces, with a valid sample of 8,148 (56.14% from towns and cities, 45.16% male, mean age 51.60 years, mean years of education 9.20 years, 73.87% married) [23]. After data cleaning, this study ultimately obtained 2,514 valid samples. The database counted the basic information of the residents in the years they covered, and the variables related to the study of this paper broadly included: age, gender, hukou registration status (China’s household registration system, which classifies individuals into urban and rural hukou based on their place of residence), socio-economic status, education level, marital status, health status, chronic disease status, physical activity and so on.

### Variables

#### Dependent variables.

The dependent variable in this study is the SRH indicator. SRH was popularized in the 1950s through social surveys, and was theoretically developed by researchers like Edward A. Suchman in 1958, who argued that it provides a comprehensive picture of the body’s subjective perceptions and objective bodily functions to obtain an individual’s health status [24]. It refers to the respondents’ overall evaluation of their own health status based on the comprehensive situation of their physical, psychological and social functions. Domestic and foreign studies believe that SRH is a comprehensive indicator reflecting the objective health status and subjective psychological expectations of the population, which can not only reflect the objective physical condition of an individual in a certain period of time, but also reflect the subjective evaluation of the individual’s own health expectations, the accessibility of health care in the living environment, etc. It is an individual’s evaluation of his or her own objective health status based on self-feelings, and compared with the traditional health indicators, it is able to more effectively reflect the health status of the population [25]. In the 2021 CGSS survey, there was a question related to SRH, “What is your current health status?” with responses categorised as very unhealthy, relatively unhealthy, average, relatively healthy, and very healthy. Research has shown that the binary classification of self-rated health (SRH) can better reflect an individual’s overall health status, and it can more clearly reveal health disparities, improve data reliability, reduce subjective bias, and enhance the stability of statistical modeling [7,26–28]. Therefore, we assigned a value of 1 to the answer “relatively healthy” and “very healthy”, which means that the health condition is good. Responses of “very unhealthy”, “relatively unhealthy”, and “average” were assigned a value of 0, representing poor health.

#### Independent variables.

In this study, chronic disease was the independent variable. In the CGSS, respondents were asked to answer whether they had a chronic disease or had a long-term health problem, and their responses were categorised as “yes” = 1 and “no” = 0.

#### Covariates.

A large body of evidence suggests that disparities in SRH among different residents are related to factors such as gender, age, marital status, socioeconomic status, educational level, insurance status, lifestyle, and related factors [29–38]. Therefore, three types of covariates related to SRH were selected for this study. The first type of covariate describes the demographic characteristics of the population, including age (continuous variable), sex (1 = male; 0 = female), household registration status (1 = urban; 0 = rural), and marital status (1 = married; 0 = other). The second type of covariate describes residents’ socioeconomic status (continuous variable), educational status (1 = high school and above; 0 = middle school and below), and insurance status (1 = yes; 0 = no). The third type of covariate describes the health-related behaviours of the population, including smoking (1 = yes; 0 = no), drinking (1 = yes; 0 = no), physical activity (1 = yes; 0 = no), sleep duration (1 = ≥7,  ≤ 9; 0 = < 7,  > 9), and sleep quality (1 = good; 0 = poor).

### Statistical analyses

Multiple data analysis methods were performed to address each of our research questions. Descriptive analyses were used to describe the mean and distribution of the variables. Two-sample t-tests were used for continuous variables and chi-square tests for categorical variables to test for disparities between urban and rural groups, and the results are displayed in Table 1. In addition, listwise deletion was used to handle missing data, which involves excluding all observations with missing data, ensuring the simplicity and accuracy of the analysis results. This method is suitable for cases where the missing data is missing completely at random (MCAR) [39–41]. In order to investigate whether and to what extent chronic diseases are associated with the SRH of the residents, logistic regression for binary SRH were employed, the Linear coefficients and SRH odds ratios (OR) with 95% confidence intervals were reported. The model is as follows:

**Table 1:** Descriptive and bivariate analyses of dependent and independent variables.

Variable	Urban (N = 760)	Rural (N = 1754)	All (N = 2514)	T-test/Chi-square Test
Self-rates health (*%*)				15.08***
Good	58.82	50.40	52.94	
Poor	41.18	49.60	47.06	
Chronic diseases (*%*)				11.27***
Yes	32.89	39.97	37.83	
No	67.11	60.03	62.17	
Age [mean (sd)]	50.81 (18.24)	52.77 (16.88)	52.18 (17.32)	2.60**
socio-economic status [mean (sd)]	2.50 (0.86)	2.20 (0.89)	2.29 (0.89)	-7.59***
Gender (*%*)				3.63
Male	48.42	44.30	45.54	
Female	51.58	55.70	54.46	
Marital status (*%*)				8.31**
Married	72.37	77.71	76.09	
Else	27.63	22.29	23.91	
Education (*%*)				182.74***
Junior high school and below	30.92	60.26	51.39	
High school and above	69.08	39.74	48.61	
physical activity (*%*)				117.60***
Yes	80.13	57.58	64.40	
No	19.87	42.42	35.60	
Smoking (*%*)				2.92
Yes	20.66	23.77	22.83	
No	79.34	76.23	77.17	
Drinking (%)				27.48***
Yes	46.32	35.23	38.58	
No	53.68	64.77	61.42	
Sleep duration (%)				4.64*
≥7, ≤ 9	67.37	62.88	64.24	
<7, >9	32.63	37.12	35.76	
Sleep quality (%)				5.41*
Good	76.58	72.12	73.47	
Poor	23.42	27.88	26.53	
Medical insurance (*%*)				5.28*
Yes	96.71	94.58	95.23	
No	3.29	5.42	4.77	
pension insurance (%)				29.69***
Yes	81.84	71.55	74.66	
No	18.16	28.45	25.34	

Note: 1. Mean or percentage were presented. 2.Standard deviation in parentheses.

* p < 0.05, ** p < 0.01, *** p < 0.001.


$$\begin{array}{c} Yu\;=\;F(Xu\beta u);\;Yr\;=\;F\left(Xr\beta r\right) \end{array}$$ (1)

Finally, the contribution of chronic disease was further explored by applying Fairlie’s non-linear decomposition (for urban-rural disparities in SRH) [42]. The model is as follows:


$$\overset{―}{Yu}-\overset{―}{Yr}=\left[\sum_{i=1}^{N^{u}}\frac{F\left(X_{i}^{u}\beta^{u}\right)}{N^{u}}-\sum_{i=1}^{N^{r}}\frac{F\left(X_{i}^{r}\beta^{u}\right)}{N^{r}}\right]+\left[\sum_{i=1}^{N^{r}}\frac{F\left(X_{i}^{r}\beta^{u}\right)}{N^{u}}-\sum_{i=1}^{N^{r}}\frac{F\left(X_{i}^{r}\beta^{r}\right)}{N^{r}}\right]$$ (2)

Some studies have shown that the Fairlie decomposition based on logistic regression model can solve the probability decomposition problem of the binary choice model, and can clearly portray the contribution of each factor to the urban-rural disparities in residents’ SRH [43]. Fairlie decomposition can be done by analysing the factors influencing the disparities in SRH between urban and rural residents and decomposing the disparities into explainable and unexplainable components. The explainable part is mainly caused by disparities in the characteristics of urban and rural residents, also known as the characteristics effect; the unexplainable part is mainly caused by unobservable factors, i.e., disparities due to urban-rural attributes when all explanatory variables are the same in urban and rural areas, also known as coefficient effects [44]. P < 0.05 was used to identify significant data in the analysis results. All statistical analyses were conducted using STATA 18.

## Results

### Descriptive statistical analysis

Table 1 showed descriptive statistics for all variables. There are disparities in the SRH of urban and rural inhabitants, 58.82%, 50.40% and 52.49% of urban, rural and all inhabitants, respectively, had a good SRH. This indicated that the overall SRH of rural residents was worse compared to urban residents. In addition, there was a significant disparity between the chronic disease status of urban and rural residents (p < 0.001), with rural residents suffering from a higher incidence of chronic disease than urban residents. The results of the t-test showed that there was a significant disparity between the socio-economic status of urban and rural residents (p < 0.001), and the mean value of urban residents was higher than that of rural residents, which indicated that the socio-economic status of urban residents was higher than that of rural residents. The results of the chi-square test showed that rural residents were more likely to have less education, with 60.26% of rural residents having junior high school or less education, almost twice as much as urban residents (30.92%). Physical activity was higher among urban residents than rural residents (p < 0.001). In addition, there were significant disparities between urban and rural residents for all variables except gender and smoking status.

### Regression analysis

Regression analyses examined independent associations between chronic diseases and residents’ SRH, and the results were shown in Table 2.

**Table 2:** Logistic regression analysis of the relationship between chronic diseases and self-rated health.

Variable	Self-rated health (Odds Ratios)		
	Urban (N = 760)	Rural (N = 1754)	All (N = 2514)
Chronic disease	0.205*** [0.140,0.300]	0.167*** [0.130,0.215]	0.179*** [0.145,0.220]
Age	0.978*** [0.966,0.989]	0.976*** [0.968,0.984]	0.976*** [0.970,0.983]
Gender	1.128 [0.757,1.679]	1.257 [0.953,1.659]	1.209 [0.964,1.516]
Marital status	0.629* [0.419,0.945]	0.917 [0.688,1.223]	0.802 [0.636,1.011]
Education	0.913 [0.620,1.345]	1.019 [0.801,1.297]	0.976 [0.797,1.195]
Socio-economic status	1.462*** [1.193,1.791]	1.467*** [1.288,1.671]	1.467*** [1.315,1.636]
physical activity	1.662* [1.088,2.538]	1.154 [0.912,1.460]	1.260* [1.028,1.545]
Smoking	0.786 [0.493,1.255]	1.058 [0.778,1.439]	0.980 [0.758,1.267]
Drinking	1.154 [0.793,1.680]	0.948 [0.726,1.238]	0.996 [0.801,1.239]
Sleep duration	1.047 [0.713,1.537]	1.161 [0.915,1.475]	1.120 [0.915,1.369]
Sleep quality	1.915*** [1.287,2.850]	2.106*** [1.623,2.734]	2.034*** [1.639,2.525]
Medical insurance	1.445 [0.532,3.925]	0.812 [0.495,1.331]	0.889 [0.570,1.386]
Pension insurance	0.911 [0.563,1.473]	0.993 [0.760,1.298]	0.986 [0.782,1.243]
Constant	1.305 [0.358,4.761]	1.790 [0.839,3.818]	1.809 [0.953,3.433]

Note: Confidence intervals are in square brackets.

* p < 0.05, ** p < 0.01, *** p < 0.001.

In the regression results, the OR values for chronic diseases were significantly less than 1 for both urban (OR=0.205) and rural (OR=0.167) residents, suggesting that increased chronic disease conditions were largely significantly associated with lower odds of improving SRH (p < 0.001). In addition, age (p < 0.001), marital status (p < 0.05), socio-economic status (p < 0.001), physical activity (p < 0.05), and sleep quality (p < 0.001) were significantly associated with SRH among urban residents. Age (p < 0.001), socio-economic status (p < 0.001), sleep quality (p < 0.001) were significantly associated with SRH among rural residents. In contrast, the regression results showed that there is no statistical significance for urban residents in terms of gender, education, smoking, drinking, sleep duration, and insurance status. Rural residents were not statistically significant in terms of gender, marital status, education, physical activity, smoking, drinking, sleep duration, and insurance status.

### Decomposition analyses

Through decomposition, this study further revealed the contribution of different factors to the disparities in SRH between urban and rural residents. The results of Fairlie’s decomposition were presented in Table 3.

**Table 3:** Fairlie decomposition analysis of disparity in self-rated healt between urban and rural residents.

Fairlie decomposition for each variable	Coefficient (95% CI)	Robust standard error	Contribution (%)
Chronic disease	-0.029*** [-0.032, -0.025]	0.002	38.915
Age	-0.007*** [-0.010, -0.005]	0.001	10.026
Gender	-0.001 [-0.003, 0.000]	0.001	1.817
Marital status	-0.001 [-0.002, 0.000]	0.001	1.298
Education	0.001 [-0.010, 0.011]	0.005	-0.708
Socio-economic status	-0.021*** [-0.027, -0.015]	0.003	28.280
physical activity	-0.010* [-0.018, -0.001]	0.004	13.277
Smoking	-0.000 [-0.001, 0.001]	0.001	0.139
Drinking	-0.000 [-0.005, 0.004]	0.002	0.035
Sleep duration	-0.001 [-0.002, 0.001]	0.001	1.146
Sleep quality	-0.004*** [-0.006, -0.003]	0.001	5.915
Medical insurance	0.001 [-0.002, 0.003]	0.001	-0.719
Pension insurance	0.000 [-0.004, 0.005]	0.002	-0.175
Explained	-0.074	—	87.851
Unexplained	-0.011	—	12.149
Difference	-0.084	—	100.00

Note: The ordering of variables was randomized in Fairlie decomposition.

* p < 0.05, ** p < 0.01, *** p < 0.001.

The results of the Fairlie decomposition showed that the proportion of the explainable part of the total disparities were 87.851% and the proportion of the unexplainable part were 12.149%, i.e., 87.851% of the urban-rural disparities in the SRH of the Chinese residents were due to the observable factors, and the other 12.149% were related to the unexplainable part (coefficient effect). The results of the decomposition of the factors showed that the disparity between chronic diseases on the SRH of urban and rural residents was statistically significant (β = -0.029, p < 0.001), which contributed the most, accounting for 38.915% of the total; In addition, age (β = -0.007, p < 0.001), socioeconomic status (β = -0.021, p < 0.001), physical activity (β = -0.010, p < 0.05), and sleep quality (β = -0.004, p < 0.001) were statistically significant in the disparity between the SRH of the urban and rural residents, with a contribution of 10.026%, 28.280%, 13.277%, and 5.915%; all other factors were not statistically significant (p > 0.05).

## Discussion

This study used data from the CGSS 2021, focused on the disparities in SRH between urban and rural residents in China, and quantified the extent to which chronic diseases explained the disparities in SRH between urban and rural residents in China. The results of descriptive, regression analyses confirmed that the SRH rate of urban residents (58.82%) was higher than that of rural residents (50.40%), which is consistent with the findings of Liu Yehong et al. [45] and Wang Yijie et al. [46], indicating that the SRH of urban residents was better than that of rural residents. Urban residents were less likely to suffer from chronic diseases (32.89%) than rural residents (39.97%), and an increase in the prevalence of chronic diseases was largely significantly associated with a decrease in the odds of improving SRH. With this in mind, we turned our attention to explaining the urban-rural disparities in SRH by conducting decomposition analyses.

The results of the study showed that 87.851% of the disparity in SRH rates between urban and rural residents was due to observed influencing factors, including chronic diseases, age, socio-economic status, physical activity and sleep quality. Among them, the disparity in the SRH rate of urban and rural residents due to chronic diseases is statistically significant (p < 0.001), and the disparity in the SRH of urban and rural residents with a contribution rate of 38.915% could be explained by the urban-rural disparity in chronic diseases, i.e., the disparity in the SRH rate between urban and rural areas decreases by 38.915% when the urban and rural areas are equally probable to suffer from the same condition of chronic diseases, indicating that the chronic disease disparity was the most important factor influencing the disparity in SRH between urban and rural residents. This phenomenon reflected the rural population’s lack of awareness of chronic diseases and their weak self-management capacity, which is consistent with the findings of scholars such as Lu Jiehua and Sun Yang [47].

Specifically, the problem of low accessibility to basic health services in rural areas may lie behind this discrepancy. The relative scarcity of medical resources and the limited level of medical services in rural areas have led to poorer management and control of chronic diseases in rural areas compared to urban areas, which is consistent with the findings of scholars such as Vicky et al [48]. Some scholars have pointed out that the primary healthcare management of non-communicable diseases in rural areas largely relies on grassroots doctors, who have received limited medical education and training [49]. In 2012, 84% of rural doctors in China did not hold a university degree, compared to 60% in urban areas [50]. At the same time, there was also a disparity between urban and rural residents in terms of health awareness, health literacy, and medical behavior. The rural residents have insufficient awareness of healthy lifestyles and behaviors, and their failure to develop good habits and healthy lifestyles not only increased their risk of illness, but also reduced their SRH [51]. On the contrary, urban residents were better able to manage chronic diseases due to more abundant healthcare resources and greater health awareness, thus improving their SRH. In addition, previous studies had shown that people with different health levels have different needs for healthcare [52]. According to some scholars’ research, the results of the study that most of the urban residents had significantly higher levels of health knowledge, attitude holding and behaviour than the rural residents also corroborated the findings of this study [53,54]. Therefore, reducing the health disparity between urban and rural residents required attention to the impact of chronic diseases. Through the implementation of chronic disease prevention and treatment, it was possible to enhance rural residents’ knowledge of chronic diseases, improve their ability to self-manage chronic diseases, advocate for rural residents to change unhealthy lifestyles and reduce health disparities between urban and rural residents, thereby promoting health equity between urban and rural residents.

Firstly, the construction of basic health services in rural areas has been strengthened, and the quality of medical services has been upgraded by raising the level of medical resource allocation in rural areas and improving the conditions of medical services. At the same time, rural health education and publicity have been strengthened to raise rural residents’ health literacy and awareness of chronic disease prevention and treatment. Secondly, chronic disease prevention and treatment have been promoted, and the early detection and treatment rates of chronic diseases have been increased through the establishment and improvement of chronic disease prevention and treatment systems and the strengthening of chronic disease monitoring and screening. At the same time, health management and follow-up work for chronic disease patients has been strengthened to enhance their self-management capabilities. Thirdly, healthy lifestyles are being promoted, and urban and rural residents (especially in rural areas) are being encouraged to take an active part in health activities by stepping up publicity and promotion of healthy lifestyles. Furthermore, to narrow the disparity between urban and rural areas, measures such as promoting telemedicine services, providing subsidies for chronic disease management to alleviate the financial burden on rural residents, improving their health security, and dispatching community health workers to underserved areas can help reduce disparities in medical resources, economic conditions, and social support. The standard of living and health protection of rural residents has been raised, thereby narrowing the disparity between urban and rural residents in terms of chronic diseases and SRH status.

Urban-rural disparities in age, socio-economic status, physical activity, and sleep quality were also important factors associated with urban-rural disparities in the SRH of the residents. Disparities in the socio-economic status of urban and rural residents are associated with 28.280% of the disparity in SRH between urban and rural areas. It indicates that the higher the socio-economic status of Chinese residents, the more positive their SRH tends to be. This was because higher socio-economic status leads to increased spending on diet and health, which in turn improves one’s health. There were great disparities in socioeconomic status and health service utilization between urban and rural residents in China [55]. For example, the elderly living in living in rural areas often do not afford medical care and were less likely to use hospitalization services [56].

This study had several limitations. First, our choice of SRH as a measure of health status, although widely validated, still had the disadvantage of subjectivity, which may pose a threat to the accuracy of health status. Second, this study only established a association, not a causal relationship, between chronic diseases and disparities in SRH between urban and rural residents. Due to the use of cross-sectional data in this study, it is not possible to rule out the influence of potential confounding factors and reverse causality. Therefore, although our study shows that chronic disease differences play an important role in the urban-rural disparity in SRH, we cannot conclude that chronic diseases directly cause these disparities. Third, Unobservable cultural factors may have an impact on the results of the analyses in this study and the generalizability of the results. Due to the unobservability of cultural factors and the inherent complexity of urban-rural health disparities, the use of cross-sectional data may not capture all relevant factors. In the future, research should collect more data for further validation, while rigorous clinical diagnostic techniques, well-designed longitudinal surveys, and experimental analyses under ethical approval will help address these limitations.

## Conclusion

Based on the results of regression and decomposition analyses, rural residents have poorer SRH and a higher prevalence of chronic diseases compared to urban residents. The increase in chronic diseases significantly reduces the likelihood of improved SRH, making the rural-urban disparity in chronic diseases a primary factor in explaining SRH disparities. Additionally, age, socioeconomic status, social participation, and sleep quality also contribute to these disparities. It is recommended to strengthen health education, emphasize disease prevention and health behavior guidance, and focus on low-education populations, labor force groups, and underdeveloped regions. Furthermore, 12.149% of the SRH disparity remains unexplained, likely due to inherent rural-urban disparities. Therefore, breaking the urban-rural divide is crucial for narrowing health disparities.

## References

[1] Fei TA. Building a healthy China: The development process and experience of China’s medical and health services under the leadership of the CPC. Manag World. 2021;37(11):26–40. doi: 10.19744/j.cnki.11-1235/f.2021.0169

[2] Li H, Yu W. Effects of the government’s health expenditure on the health of rural residents in China. Soc Sci China. 2013;10:41–60.

[3] Bai M. The current situation, problems and countermeasures of healthy village construction in the context of rural revitalization. Rural Econ. 2020;7:119–26.

[4] National Health Commission (NHC). Monitoring of Health Literacy Among Chinese Residents in 2023. 2024 Available from: https://www.gov.cn/lianbo/bumen/202404/content6947232.htm

[5] Downey N, DiBenedetto J. Health disparity concept analysis. Diversity Equal Health Care. 2021;18(1):224–31.

[6] McCartney G, Popham F, McMaster R, Cumbers A. Defining health and health inequalities. Public Health. 2019;172:22–30. doi: 10.1016/j.puhe.2019.03.023 31154234 PMC6558275

[7] Idler EL, Benyamini Y. Self-rated health and mortality: a review of twenty-seven community studies. J Health Soc Behav. 1997;38(1):21–37. 9097506

[8] Roberts ME, Doogan NJ, Kurti AN, Redner R, Gaalema DE, Stanton CA, et al. Rural tobacco use across the United States: How rural and urban areas differ, broken down by census regions and divisions. Health Place. 2016;39:153–9. doi: 10.1016/j.healthplace.2016.04.001 27107746 PMC4874850

[9] Chan GCK, Leung J, Quinn C, Kelly AB, Connor JP, Weier M, et al. Rural and Urban Differences in Adolescent Alcohol Use, Alcohol Supply, and Parental Drinking. J Rural Health. 2016;32(3):280–6. doi: 10.1111/jrh.12151 26450773

[10] Anderson TJ, Saman DM, Lipsky MS, Lutfiyya MN. A cross-sectional study on health differences between rural and non-rural U.S. counties using the County Health Rankings. BMC Health Serv Res. 2015;15:441. doi: 10.1186/s12913-015-1053-3 26423746 PMC4590732

[11] Arruda NM, Maia AG, Alves LC. Inequality in access to health services between urban and rural areas in Brazil: a disaggregation of factors from 1998 to 2008. Cad Saude Publica. 2018;34(6):e00213816. doi: 10.1590/0102-311X00213816 29947662

[12] Kaczmarek M, Pacholska-Bogalska J, Kwaśniewski W, Kotarski J, Halerz-Nowakowska B, Goździcka-Józefiak A. The association between socioeconomic status and health-related quality of life among Polish postmenopausal women from urban and rural communities. Homo. 2017;68(1):42–50. doi: 10.1016/j.jchb.2016.11.004 28024658

[13] Ryan-Nicholls KD. Health and sustainability of rural communities. Rural Remote Health. 2004;4(1):242. 15882108

[14] Bonnell LN, Clifton J, Rose GL, Waddell EN, Littenberg B. Urban-Rural Differences in Mental and Physical Health among Primary Care Patients with Multiple Chronic Conditions: A Secondary Analysis from a Randomized Clinical Trial. Int J Environ Res Public Health. 2022;19(23):15580. doi: 10.3390/ijerph192315580 36497657 PMC9741371

[15] Westaway MS. The impact of chronic diseases on the health and well-being of South Africans in early and later old age. Arch Gerontol Geriatr. 2010;50(2):213–21. doi: 10.1016/j.archger.2009.03.012 19398135

[16] Molarius A, Janson S. Self-rated health, chronic diseases, and symptoms among middle-aged and elderly men and women. J Clin Epidemiol. 2002;55(4):364–70. doi: 10.1016/s0895-4356(01)00491-7 11927204

[17] Barreto SM, Figueiredo RC de. Chronic diseases, self-perceived health status and health risk behaviors: gender differences. Rev Saude Publica. 2009;43 Suppl 2:38–47. doi: 10.1590/s0034-89102009000900006 19936497

[18] Wolff JL, Starfield B, Anderson G. Prevalence, expenditures, and complications of multiple chronic conditions in the elderly. Arch Intern Med. 2002;162(20):2269–76. doi: 10.1001/archinte.162.20.2269 12418941

[19] Fu S, Huang N, Chou Y-J. Trends in the prevalence of multiple chronic conditions in Taiwan from 2000 to 2010: a population-based study. Prev Chronic Dis. 2014;11:E187. doi: 10.5888/pcd11.140205 25340359 PMC4208993

[20] General Office of the State Council. Circular of the General Office of the State Council on the Issuance of the Medium- and Long-Term Plan for thePrevention and Treatment of Chronic Diseases in China (2017-2025). 2017. Available from: http://www.gov.cn/zhengce/content/2017-02/14/content_5167886.Htm

[21] Hu Y, Yang Y, Gao Y, Zhao L, Chen L, Sui W, et al. The impact of chronic diseases on the health-related quality of life of middle-aged and older adults: the role of physical activity and degree of digitization. BMC Public Health. 2024;24(1):2335. doi: 10.1186/s12889-024-19833-8 39198736 PMC11351089

[22] Wang XY. Construction and validation of the middle-range theory of chronic disease adaptation. Zhejiang University; 2020. doi: 10.27461/d.cnki.gzjdx.2020.002292

[23] Zhu W, Liu S, Wang R. Impact of internet use on physical and mental health of rural residents: empirical analysis based on CGSS2021 data. J Sichuan Agr Univ. 2024;42(3):689–97. doi: 10.16036/j.issn.1000-2650.202402361

[24] Jylhä M. What is self-rated health and why does it predict mortality? Towards a unified conceptual model. Soc Sci Med. 2009;69(3):307–16. doi: 10.1016/j.socscimed.2009.05.013 19520474

[25] Miilunpalo S, Vuori I, Oja P, Pasanen M, Urponen H. Self-rated health status as a health measure: the predictive value of self-reported health status on the use of physician services and on mortality in the working-age population. J Clin Epidemiol. 1997;50(5):517–28. doi: 10.1016/s0895-4356(97)00045-0 9180644

[26] Zajacova A, Dowd JB. Reliability of self-rated health in US adults. Am J Epidemiol. 2011;174(8):977–83. doi: 10.1093/aje/kwr204 21890836 PMC3218632

[27] Lu W, Lu Z, Huang Y, Wu X, Fu T, Zhang J. Association of adverse childhood experiences with self-rated health among Chinese elderly people. Chin Gen Pract. 2022;25(25):3101–6. doi: 10.12114/j.issn.1007-9572.2022.0379

[28] Zhuang Z, Zhang Y, Li C, Wang Z, Lv Q. The impact of self-rated health and self-care ability and their interaction on depression symptoms in the elderly. Mod Prev Med. 2025;52(3):497–502. doi: 10.20043/j.cnki.MPM.202408051

[29] Olshansky SJ, Ault AB. The fourth stage of the epidemiologic transition: the age of delayed degenerative diseases. Milbank Q. 1986;64(3):355–91. 3762504

[30] Fuhrer R, Stansfeld SA. How gender affects patterns of social relations and their impact on health: a comparison of one or multiple sources of support from “close persons”. Soc Sci Med. 2002;54(5):811–25. doi: 10.1016/s0277-9536(01)00111-3 11999495

[31] Kawachi I, Berkman LF. Social Cohesion, Social Capital, and Health. In: Social Epidemiology. Oxford University Press. 2000. p. 174–190.

[32] Lowry D, Xie Y. Socioeconomic Status and Health Differentials in China: Convergence or Divergence at Old Ages. Population Studies Center, University of Michigan. 2009.

[33] Chen L, Yip W, Chang M-C, Lin H-S, Lee S-D, Chiu Y-L, et al. The effects of Taiwan’s National Health Insurance on access and health status of the elderly. Health Econ. 2007;16(3):223–42. doi: 10.1002/hec.1160 16929478

[34] Chelak K, Chakole S. The Role of Social Determinants of Health in Promoting Health Equality: A Narrative Review. Cureus. 2023;15(1):e33425. doi: 10.7759/cureus.33425 36751221 PMC9899154

[35] Boffetta P, Hashibe M. Alcohol and cancer. Lancet Oncol. 2006;7(2):149–56. doi: 10.1016/S1470-2045(06)70577-0 16455479

[36] Kang H-R, Kim SJ, Nam JG, Park YS, Lee C-H. Impact of Smoking and Chronic Obstructive Pulmonary Disease on All-Cause, Respiratory, and Cardio-Cerebrovascular Mortality. Int J Chron Obstruct Pulmon Dis. 2024;19:1261–72. doi: 10.2147/COPD.S458356 38863653 PMC11166149

[37] Warburton DER, Nicol CW, Bredin SSD. Health benefits of physical activity: the evidence. CMAJ. 2006;174(6):801–9. doi: 10.1503/cmaj.051351 16534088 PMC1402378

[38] Weighall A, Kellar I. Sleep and memory consolidation in healthy, neurotypical children, and adults: a summary of systematic reviews and meta-analyses. Emerg Top Life Sci. 2023;7(5):513–24. doi: 10.1042/ETLS20230110 39288097

[39] Little RJ, Rubin DB. Statistical Analysis with Missing Data. 2nd ed. Hoboken, New Jersey: John Wiley & Sons, Inc; 2002.

[40] Enders CK. Applied Missing Data Analysis. Guilford Press; 2010.

[41] Graham JW. Missing data analysis: making it work in the real world. Annu Rev Psychol. 2009;60:549–76. doi: 10.1146/annurev.psych.58.110405.085530 18652544

[42] Fairlie RW. An extension of the Blinder-Oaxaca decomposition technique to logit and probit models. J Econ Soc Meas. 2005;30(4):305–16. doi: 10.3233/JEM-2005-0259

[43] Cai C, Xu Q. Research on urban-rural difference of higher education opportunities: empirical results through O-B decomposition of binary choice. Statist Inf Forum. 2012;27(4):87–93. doi: 10.3969/j.issn.1007-3116.2012.04.015

[44] Xie E. Analysis of regional differences in health in China. J Shanxi Finance Econ Univ. 2011;33(8):11–24. doi: 10.13781/j.cnki.1007-9556.2011.08.004

[45] Liu YH, Guo HD, Shi XJ. Measurement and trend analysis of health opportunity inequality among urban and rural residents in China. Popul Dev. 2023;29(6):72–87.

[46] Wang YJ, Cheng P. Different influence of socioeconomic status on urban and rural residents’ health. J Northwest A & F Univ (Soc Sci Ed). 2015;15(6):117–23. doi: 10.13968/j.cnki.1009-9107.2015.06.017

[47] Lu J, Sun Y. Elderly health in rural China: characteristics, causes and responding strategies. J China Agric Univ (Soc Sci). 2024;41(2):49–67. doi: 10.13240/j.cnki.caujsse.2024.02.005

[48] Qin VM, McPake B, Raban MZ, Cowling TE, Alshamsan R, Chia KS, et al. Rural and urban differences in health system performance among older Chinese adults: cross-sectional analysis of a national sample. BMC Health Serv Res. 2020;20(1):372. doi: 10.1186/s12913-020-05194-6 32366235 PMC7197140

[49] Ding H, Chen Y, Yu M, Zhong J, Hu R, Chen X, et al. The Effects of Chronic Disease Management in Primary Health Care: Evidence from Rural China. J Health Econ. 2021;80:102539. doi: 10.1016/j.jhealeco.2021.102539 34740053

[50] Meng Q, Yang H, Chen W, Sun Q, Liu X. People’s Republic of China health system review. World Health Organization. 2015. Available from: https://apo.who.int/publications/i/item/9789290617280

[51] Wang W, Zhang Y, Lin B, Mei Y, Ping Z, Zhang Z. The Urban-Rural Disparity in the Status and Risk Factors of Health Literacy: A Cross-Sectional Survey in Central China. Int J Environ Res Public Health. 2020;17(11):3848. doi: 10.3390/ijerph17113848 32485790 PMC7312746

[52] Asmus-Szepesi KJE, de Vreede PL, Flinterman LE, Nieboer AP, Bakker TJEM, Borsboom GJJM, et al. Prognosis of hospitalised older people with different levels of functioning: a prospective cohort study. Age Ageing. 2013;42(6):803–9. doi: 10.1093/ageing/aft126 23974209

[53] Li S, Zhang R, Li Y, Wang Y, Zou Q. Efficacy of China healthy lifestyle for all among urban and rural residents in Liaoning province. Chin J Public Health. 2017;33(12):1693–6. doi: 10.11847/zgggws2017-33-12-05

[54] Liu Y, Wang S, Jiang B. Analysis on influence factors and countermeasures of old people’s health in Siping city of Jilin province. Popul J. 2010;2:28–34. doi: 10.3969/j.issn.1004-129X.2010.02.005

[55] Li Y, Chi I, Zhang K, Guo P. Comparison of health services use by Chinese urban and rural older adults in Yunnan province. Geriatr Gerontol. 2010;6(4):260–9. doi: 10.1111/j.1447-0594.2006.00358.x

[56] Shi L. Health care in China: a rural-urban comparison after the socioeconomic reforms. Bull World Health Organ. 1993;71(6):723–36. 8313490 PMC2393531

